# Manipulating reproductive effort leads to changes in female reproductive scheduling but not oxidative stress

**DOI:** 10.1002/ece3.786

**Published:** 2013-09-25

**Authors:** Edith D Aloise King, Michael Garratt, Robert Brooks

**Affiliations:** Evolution & Ecology Research Centre and School of Biological, Earth and Environmental Sciences, University of New South WalesSydney, NSW, 2052, Australia

**Keywords:** Ageing, aging, cost of reproduction, cross-foster, Free Radical Theory of ageing, life history, oxidative damage, postpartum pregnancy, senescence, trade-off

## Abstract

The trade-off between reproductive investment and lifespan is the single most important concept in life-history theory. A variety of sources of evidence support the existence of this trade-off, but the physiological costs of reproduction that underlie this relationship remain poorly understood. The Free Radical Theory of Ageing suggests that oxidative stress, which occurs when there is an imbalance between the production of damaging Reactive Oxygen Species (ROS) and protective antioxidants, may be an important mediator of this trade-off. We sought to test this theory by manipulating the reproductive investment of female mice (*Mus musculus domesticus*) and measuring the effects on a number of life history and oxidative stress variables. Females with a greater reproductive load showed no consistent increase in oxidative damage above females who had a smaller reproductive load. The groups differed, however, in their food consumption, reproductive scheduling and mean offspring mass. Of particular note, females with a very high reproductive load delayed blastocyst implantation of their second litter, potentially mitigating the costs of energetically costly reproductive periods. Our results highlight that females use strategies to offset particularly costly periods of reproduction and illustrate the absence of a simple relationship between oxidative stress and reproduction.

## Introduction

The life history strategy of an organism is governed by a co-adapted set of traits that influence its pattern of reproduction and mortality (Stearns 1992). These include size at birth, growth rate, number and size of offspring and length of lifespan. Life history theory predicts that investment in these traits is costly, requiring organisms to balance their investment in different life history traits (Stearns 1992). As a consequence, organisms use a wide variety of life history strategies to increase their fitness in a given environmental niche, and individuals show further plasticity in their investment in different traits to maximise reproductive success in relation to prevailing conditions (Edward and Chapman 2011).

Reproduction is thought to be especially costly and can impact various fitness components on different levels. Ultimately, costs of reproduction refer to the reduction in residual reproductive effort as a result of reproductive investment. Future reproduction and lifespan represent the fitness components most closely tied to residual reproductive effort (Stearns 1992). The life history traits that are impacted by reproductive investment also depend on the reproductive strategy of an organism and their relative impact on that animal's fitness (Hamel et al. 2010). Shorter-lived animals, for example, are more likely to show survival costs of reproduction, not reduced future reproductive output, since their life history strategy usually favours high levels of investment in reproduction early in life (Hamel et al. 2010).

The wide-spread occurrence of these deleterious consequences of reproduction highlight the presence of proximate mechanisms that prevent organisms from investing in other traits while maximising reproductive effort (Zera and Harshman 2001). These mechanisms may manifest as physiological costs of reproduction that directly reduce investment in other life history traits, such as lifespan, or the strategic re-allocation of resources from somatic maintenance to reproduction, or may involve both of these processes acting in combination (Harshman and Zera 2007). Despite the long history of this idea, the physiological consequences of reproduction that underlie the reproduction-lifespan trade-off remain elusive.

The Free Radical Theory of Aging suggests that oxidative stress plays a key role in the aging process (Harman 1956; Beckman and Ames 1998; Monaghan et al. 2009). This theory is supported by a growing body of evidence (reviewed by Sohal and Weindruch 1996; Sohal et al. 2002), some of which implicates oxidative stress as a causal factor in a number of age-related diseases (Beckman and Ames 1998; Salmon et al. 2010). Oxidative stress is an often-pathological process that occurs when there is a serious overproduction of reactive oxygen species (ROS) in relation to defence mechanisms (Monaghan et al. 2009). This can cause damage to biomolecules with important functions (Dowling and Simmons 2009; Monaghan et al. 2009) and limit the capacity of organisms to respond to redox changes within the cellular environment (Jones 2006, 2008; Garratt and Brooks 2012). Hypotheses in this field have recently expanded to include the possibility that oxidative stress underlies a number of life-history trade-offs (Dowling and Simmons 2009; Monaghan et al. 2009; Isaksson et al. 2011; Selman et al. 2012). This addition to the classic theory stems from predictions that oxidative stress may increase during periods of elevated metabolic rate, for example during costly reproductive events (Speakman 2008; Dowling and Simmons 2009; Monaghan et al. 2009). It has been promoted, therefore, as a promising candidate in the search for the missing link between reproductive effort and senescence (Dowling and Simmons 2009; Metcalfe and Alonso-Alvarez 2010).

Recent tests of the relationship between reproductive investment and oxidative stress have produced mixed results (reviewed by Costantini 2008; Monaghan et al. 2009; Metcalfe and Alonso-Alvarez 2010; Selman et al. 2012). Studies of small mammals have demonstrated positive correlations between litter size and oxidative damage (Bergeron et al. 2011; Garratt et al. 2011) and associations between lactation (which was coupled with elevated daily energy expenditure) and oxidative damage (Fletcher et al. 2012). These results provide partial support for the hypothesis that oxidative stress increases with reproductive effort, although they do not provide direct evidence that oxidative damage is elevated with a manipulated increase in reproductive effort. In contrast to these findings, Nussey et al. (2009) showed that oxidative damage did not correlate with recent or total reproductive effort in wild female Soay sheep (*Ovis aries*) and laboratory comparisons between reproducing and non-reproducing mice (*Mus musculus domesticus*) and bank voles (*Myodes glareolus*) paradoxically suggest that reproducing animals experience less oxidative damage than virgin animals (Garratt et al. 2011; Ołdakowski et al. 2012).

One limitation of the aforementioned studies is that comparisons were made between animals allowed to reproduce and those that were not, allowing animals to control, to a degree, their relative reproductive allocation (Metcalfe and Monaghan 2013). Only one study to date has experimentally manipulated reproductive effort and measured the resulting oxidative damage. Garratt et al. (2013) cross-fostered newborn mice to produce litters of two and eight pups, and compared oxidative damage and antioxidant activity between females lactating for large and small litters (as well as non-reproductive females). Garratt et al. (2013) found no increase in oxidative damage among female mice lactating for large litters. Instead, they observed that oxidative damage to proteins in the liver was reduced among females lactating for large litters, and that these individuals also exhibited greater activity of the antioxidant enzyme superoxide dismutase in this tissue. This study provided a valuable insight, but the absence of a positive effect must be interpreted cautiously because the period of reproductive investment was brief (the subjects were sacrificed at the peak of investment in their first ever litter). Perhaps the differential of investment between these treatment groups was not adequate to reveal differences in oxidative stress? We therefore designed the current study with a more dramatic manipulation of reproductive effort over a more protracted period.

**Figure 1:**
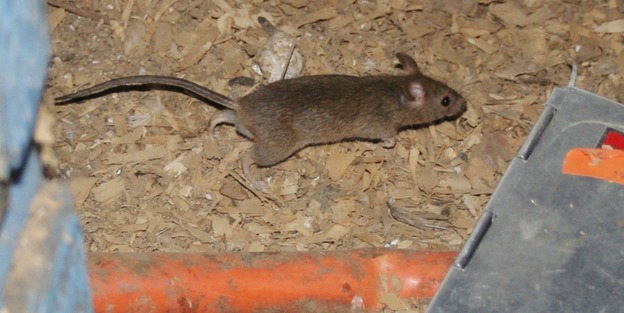
A house mouse, *Mus musculus domesticus*, from the source population from which we derived the mice used in this study.

We used a two by two factorial design to construct a gradient of reproductive effort in wild-derived mice (*Mus musculus domesticus*; Fig. 1) by manipulating litter size and the opportunity for females to mate during the postpartum estrus. Female mice, like a number of mammal species, display a short window of estrus immediately following parturition. If they conceive at this postpartum estrus, then they will be concurrently pregnant with a second litter while they are lactating for the first litter. Since the periods of lactation and gestation are of similar length in mice (approximately 21 and 19 days, respectively), the peak demands of the concurrent pregnancy tend to overlap with the peak demands of lactation (Johnson et al. 2001). Females with the highest level of reproductive allocation, therefore, were allocated a large litter of eight pups and were mated during the postpartum estrus so that they were concurrently pregnant with their second litter during the first lactation. Representing the lowest level of reproductive effort, some females were allocated a small litter of two pups and were not allowed to mate during the postpartum estrus and so were not lactating and pregnant simultanously.

When the subjects reached the peak of the second lactation (approximately 2 weeks after the second parturition) we collected tissue samples and measured several markers of oxidative damage, to test whether oxidative stress is a cost of reproductive effort. We also used this opportunity to take a more integrative approach and assess the life history costs and adaptations to these graded levels of reproductive expenditure. It is possible that animals will mitigate some of the somatic costs of reproduction and avoid oxidative stress by adjusting their reproductive allocation and scheduling to offset energetically demanding periods of reproduction. Changes in offspring mass, parturition date and energy reserves as a consequence of reproductive allocation have been termed indirect costs of reproduction (Hamel et al. 2010). As an index of maternal allocation (Ortiz et al. 1984; Ross 1988; Pontier et al. 1989) we examined the weights of offspring from each litter at weaning and also food consumption at peak-lactation. We also tested whether female allocation to their second litter is delayed in relation to the reproductive burden of their first. Early correlative studies hint that postpartum pregnant females may actually delay implantation of blastocysts when lactating for particularly large litters (Mantalenakis and Ketchel 1966; Norris and Adams 1981; Johnson et al. 2001). Our litter manipulation protocol allowed us to test for such a physiological adaptation to reproduction experimentally.

## Materials and Methods

### Subjects

Subjects (*n* = 80) were the captive-bred first generation progeny of 26 pairs of wild house mice captured in Sydney, Australia between March and May 2011. They were weaned at 4 weeks old, then housed in sibling groups until 6 weeks old, at which point the sexes were separated. Females were housed with sisters in groups of two, three or four until the beginning of the experiment, when they ranged between 10 and 18 weeks old. Males used for breeding were housed in isolation until the beginning of the experiment, when they ranged between 8 and 19 weeks old.

Cages (51 × 21 × 13 cm) were lined with corncob bedding (Shepherd's Cob, Watertown, TN) and contained tissues and shredded newspaper for nesting. Subjects had ad libitum access to water and Gordon's mouse maintenance feed (Gordon's Specialty Stockfeeds, Yanderra, Australia) throughout the experiment. This diet contained no added vitamin C, vitamin E at concentration of 40.192 iu per kg finished feed, and an antioxidant premix used to aid food preservation (Oxystat) at a finished feed rate of 832 ppm. We have previously shown that presence of this preservative has no impact on oxidative stress in reproducing female mice (Garratt et al. 2013). A full breakdown of the this diet is also displayed by Garratt et al. (2013). We chose to feed animals ad libitum because mice that are food limited do not reproduce, thus obscuring our reproductive manipulations. Further, the idea that oxidative stress moderates life-history trade-offs is often presented as an alternative to the idea that energetic limitations play this role. Subjects were maintained under a 12L:12D reverse light cycle with lights on at 19:30. All animal handling took place in the dark phase under red light.

#### Experiment

Females were randomly allocated to one of four treatment combinations (postpartum breeding, small litter; sequential breeding, small litter; postpartum breeding, large litter; and sequential breeding, large litter), with the proviso that siblings were divided between treatments wherever possible. The mean age of female subjects did not differ between treatment groups. Males were then randomly allocated to females, ensuring no full-sib or half-sib pairs.

Because the sequential breeding treatment (i.e. no postpartum mating) takes longer to apply, we split this treatment into two blocks commencing 3 weeks apart. The first half, then, finished the experiment at the same time as the postpartum breeding animals. The second half began breeding at the same time as the postpartum treatment. Two-tailed *t*-tests revealed no differences between blocks in any life history traits, and although the first block did display higher levels of urinary 8-hydroxy-2-deoxy guanosine than the second block at the peak of the second lactation (*t*_17_ = −2.23, *P* = 0.04) there were no other differences in oxidative stress between the blocks.

One week before breeding, the cages for each pair of mates were placed side-by-side to allow the male and female to familiarise. Three days before pairing, a small handful of corncob bedding and nesting material was exchanged between the cages. In the sequential breeding treatment, males were removed from the female's cage when the female was close to parturition (assessed visually by swelling of the abdomen) and were reinserted 1 week later, at least 2 days after parturition. These males were therefore absent during the female's postpartum estrus. Due to the error involved in the visual assessment of pregnancy, there was some variation in the period of male removal (the mean duration was 7.95 days). In the postpartum breeding treatment, males were present during parturition and the postpartum estrus, and removed for a period of 1 week beginning 2 days after parturition. All males were housed adjacent to their mate during the period of removal.

All pups from each female's first litter were cross-fostered at birth to produce the litter manipulation (cross-fostering methods are described below). At 28 days old, the cross-fostered litter was removed, sexed and weighed. When the dam was visibly pregnant again, the male was removed permanently and the dam was housed in isolation until the second litter was born. This litter was not manipulated. Between days 14 and 17 of the second lactation the dam was culled by cervical dislocation and immediately dissected. This point in time is when studies show that the energetic demands of lactation have reached a climax (König et al. 1988; Johnson et al. 2001), including an example from our own population of wild-derived mice (Garratt et al. 2013).

#### Urine collection

Urine samples were collected from each female at the peak of the first and second lactation. To collect urine, females were placed in an empty cage on an elevated wire grate for a maximum of two hours or until they produced at least 100 μL urine in total. Cages were checked every few minutes for urine, which was immediately collected and snap-frozen in liquid nitrogen. Samples were then stored at stored at −80°C until analysis.

#### Food consumption

Food consumption was measured over a 24 h period at 14 days post-partum of the first litter. Measurements reflected the consumption of both male and female since they were co-housed.

#### Cross-fostering

Females were checked daily for litters and when parturition was noted the litter size and total litter mass was recorded and all pups were removed from the nest. The dam was provided with either two or eight newborn pups from one, two or three donor dams (*n* = 27, 45 and 2 respectively). There was no bias in the number of donor dams between the small and large litter treatments. Most pups were cross-fostered between experimental animals but due to limitations in pup availability, some were sourced from breeding stocks (*n* = 10). On the occasions when no newborn pups were available the cross-fostering took place on the following day (*n* = 5). Excess pups were humanely culled.

#### Measurements of oxidative stress

Immediately after culling, subjects were dissected and liver and heart were snap frozen in liquid nitrogen and stored at −80°C. Tissue samples were later ground under liquid nitrogen using a mortar and pestle. Total glutathione and oxidised glutathione were measured using the automated glutathione recycling assay described by Anderson (1996), adjusted for use on a 96-well plate reader (Vasilaki et al. 2006). Protein thiols were measured using the method described by Di Monte et al. (1984), also adjusted for use on a 96-well plate reader (Vasilaki et al. 2006). DNA oxidation in urine samples was measured using Cayman Chemical's 8-hydroxy-2-deoxy guanosine ELISA kit (Ann Arbor, MI), and urinary concentrations of 8-hydroxy-2-deoxy guanosine were standardised to creatinine levels. Concentrations of creatinine were obtained using the method described by Cheetham et al. (2009).

#### Data analysis

Animals that failed to breed or failed to raise their first or second litter were removed from the experiment. In addition, females from the postpartum breeding treatment that could not confidently be categorized as postpartum pregnant (i.e. for whom the interval between the two births was greater than 30 days; Bruce and East 1956) were also removed, giving a final sample size of 36 females.

Analyses were performed in SPSS (version 20; SPSS Inc., Chicago, IL) using general linear mixed models (GLMM) with postpartum pregnancy, manipulated litter size and their interaction as fixed effects, and the identity of the dam and sire of each subject controlled for as a random effect. The subject's number of siblings was included in analyses of oxidative stress, since Garratt et al. (2013) found it to be positively correlated with some measures of oxidative stress. The subject's number of siblings, the size of the second litter and starting body mass (log transformed) were treated as covariates when included in the analyses. Urinary 8-hydroxy-2-deoxy guanosine (8-OHdG) was analysed using a repeated measures GLMM in which time (first or second lactation) was included as a fixed factor and individual identity was treated as a random factor. Non-significant terms in each model were removed by backwards selection. Food consumption data and levels of urinary 8-OHdG were not normally distributed and were log-transformed. The proportion of oxidised glutathione was not normally distributed for the liver or heart so both variables were logit transformed (Warton and Hui 2011). Data associated with this study are archived at Dryad (datadryad.org/).

We obtained anomalous values of oxidised glutathione for two animals (one for the heart and the other for the liver). Anderson (1996) reports that oxidised glutathione usually comprises less than 5% of total glutathione; the anomalous values were approximately double this concentration. Since these animals did not display high values for any other oxidative stress measure, we thought it likely that the values resulted from an error during the assay and removed them from the relevant analyses.

## Results

The mean size of the first litter (prior to cross-fostering) was 5.5 pups (± 0.16 SE) and ranged between two and eight pups. On average, dams that were allocated small litters received 3.3 (± 0.18 SE) fewer pups than their natural litter size, and dams that were allocated large litters received 2.4 (± 0.28 SE) more pups than their natural litter size.

Dams that conceive at the postpartum estrus can delay the implantation of blastocysts in the gestating litter in order to somewhat offset the most demanding periods of pregnancy and lactation (Bruce and East 1956). The extent to which females make use of this delay varies (Bruce and East 1956; Mantalenakis and Ketchel 1966), as does the length of the delay, which has been observed to positively correlate with the number of pups in the suckling litter (Bruce and East 1956; Norris and Adams 1981; Johnson et al. 2001). In general, the interval between the birth of the senior and junior litters does not exceed 36 days (Bruce and East 1956). Of the animals mated at the postpartum estrus, 20 dams (80%) successfully conceived and gave birth to their second litter within 30 days of the first parturition. Two dams (8%) displayed intervals between the first and second parturition of greater than 36 days, indicating that they probably failed to conceive at the postpartum estrus (Bruce and East 1956). The remaining three dams (12%) exhibited inter-litter intervals of 31, 33 and 34 days, which could result from postpartum matings or post-weaning matings. Due to this ambiguity, only animals that exhibited an inter-birth interval of 30 days or less were included in the analyses.

### Female physiology

**Figure 2:**
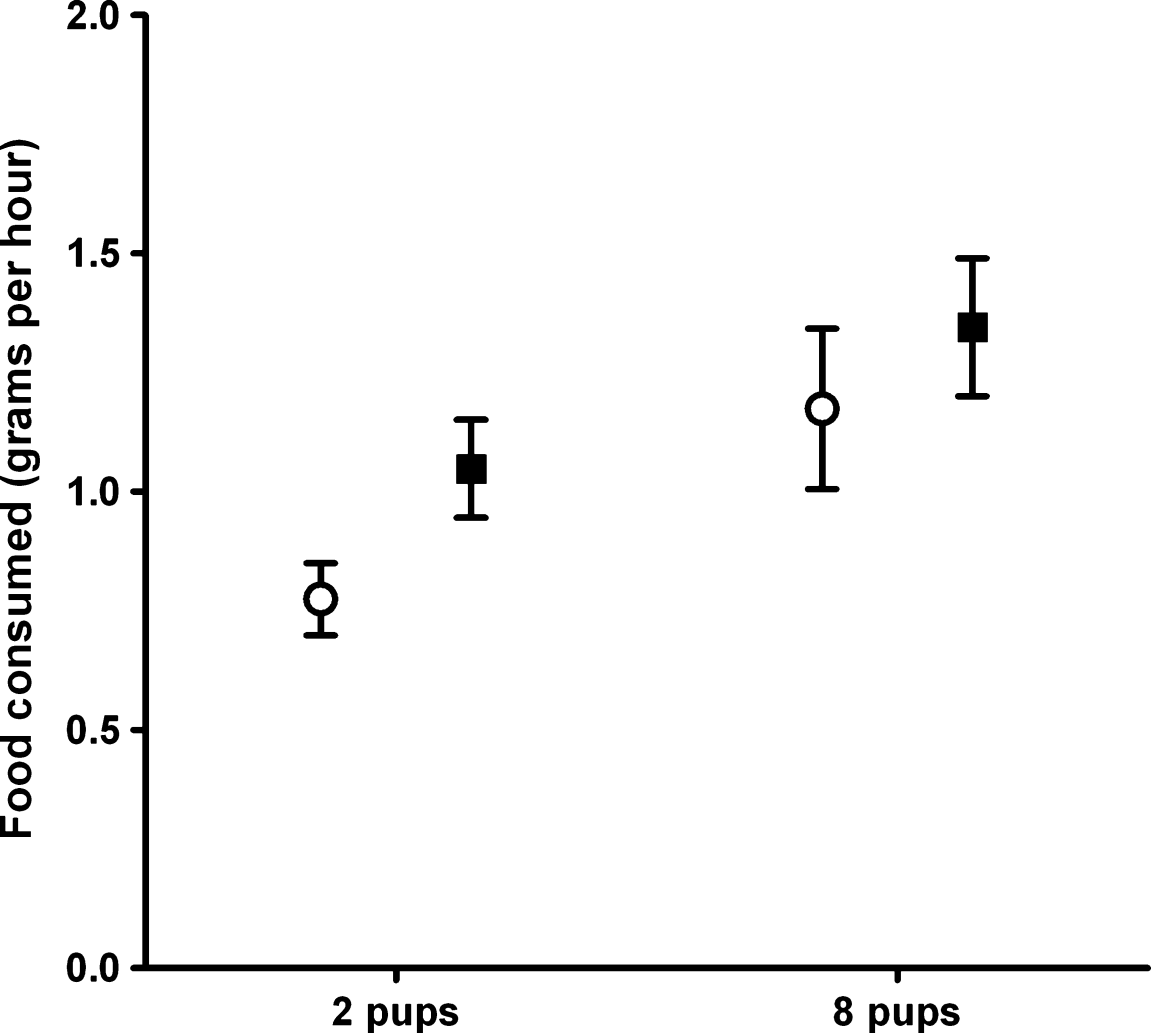
Food consumption at the peak of the first lactation increases with litter size and with postpartum pregnancy (postpartum pregnant females are represented by closed squares; non-pregnant females are represented by open circles).

Subjects exhibited differences in the volume of food consumed at peak lactation (day 14 after parturition) of the first litter. This is an important point in time, since it reflects the most energetically expensive period of lactation, and includes the added burden of concurrent pregnancy for females in the postpartum breeding treatment. It is therefore the point at which the effects of our manipulations were probably strongest. Females that were postpartum pregnant ate more food than those that were not pregnant (*F*_1, 26.6_ = 4.18, *P* = 0.05), and females lactating for large litters ate more than those that were lactating for small litters (*F*_1, 31.7_ = 7.26, *P* = 0.01; interaction term *F*_1, 30.4_ = 0.19, *P* = 0.66). In this respect, food consumption was positively correlated with the expected gradient of reproductive effort at this point in time (Fig. 2).

A multivariate analysis of variance (MANOVA) revealed that postpartum pregnancy (‘PP’) and manipulated litter size (‘MLS’) produced no clear changes to markers of oxidative stress in the liver and heart (Table 1). Individual linear mixed models for each oxidative stress variable allowed for the inclusion of relevant covariates; these also failed to reveal any convincing or consistent effects of our manipulations on any measures of oxidative stress (Table 2). The inclusion of the dam's own litter size (‘OLS’) and the size of the second litter (‘SLS’) as covariates served to reveal some significant effects of postpartum pregnancy and manipulated litter size on liver protein thiols and oxidised glutathione, respectively. But these main effects are so weak that they cannot be seen without fitting covariate by treatment interactions that were not predicted a priori and are without clear biological explanations. We are compelled, therefore, to treat the evidence as a refutation of any effect of postpartum pregnancy, litter size manipulation and their interaction on this suite of oxidative measures.

**Table 1:** MANOVA exploring the effects of postpartum pregnancy, manipulated litter size and the interaction between these treatments on measures of oxidative stress in the liver and heart

	Multivariate tests		
	
	Postpartum pregnancy	Manipulated litter size	PP × MLS
Wilks' Lambda	0.79	0.81	0.80
*F*	0.93	0.81	0.88
df	6, 21	6, 21	6, 21
*P*	0.50	0.57	0.53

	Univariate tests		
	
	df	*F*	*P*
Source and dependent variable:			
Postpartum pregnancy			
Protein thiols (liver)	1, 29	0.41	0.53
Protein thiols (heart)	1, 29	0.83	0.37
Total glutathione (liver)	1, 29	0.40	0.53
Total glutathione (heart)	1, 29	4.12	0.05
Proportion of glutathione oxidised (liver)	1, 29	1.96	0.17
Proportion of glutathione oxidised (heart)	1, 29	0.08	0.78
Manipulated litter size			
Protein thiols (liver)	1, 29	0.42	0.52
Protein thiols (heart)	1, 29	0.12	0.74
Total glutathione (liver)	1, 29	1.66	0.21
Total glutathione (heart)	1, 29	0.90	0.35
Proportion of glutathione oxidised (liver)	1, 29	3.16	0.09
Proportion of glutathione oxidised (heart)	1, 29	<0.01	0.96
PP × MLS			
Protein thiols (liver)	1, 29	0.09	0.76
Protein thiols (heart)	1, 29	2.30	0.14
Total glutathione (liver)	1, 29	0.38	0.54
Total glutathione (heart)	1, 29	3.23	0.08
Proportion of glutathione oxidised (liver)	1, 29	0.19	0.67
Proportion of glutathione oxidised (heart)	1, 29	0.38	0.54

**Table 2:** Individual GLMMs for protein thiols, total glutathione and oxidised glutathione including relevant covariates and their interactions with main effects

	Protein thiols						Total glutathione						Proportion glutathione oxidised (logit transformed)					
			
	Liver			Heart			Liver			Heart			Liver			Heart		
						
	df	*F*	*P*	df	*F*	*P*	df	*F*	*P*	df	*F*	*P*	df	*F*	*P*	df	*F*	*P*
Model																		
Postpartum pregnancy	**1, 17.9**	**6.80**	**0.02**	**1, 24.9**	**0.03**	**0.86**	**1, 27.0**	**1.50**	**0.23**	**1, 25.1**	**0.02**	**0.88**	**1, 25.5**	**1.25**	**0.27**	**1, 22.8**	**0.21**	**0.65**
Manipulated litter size	**1, 15.2**	**1.41**	**0.25**	**1, 26.0**	**1.14**	**0.30**	**1, 27.5**	**2.51**	**0.12**	**1, 26.5**	**0.30**	**0.59**	**1, 26.3**	**5.52**	**0.03**	**1, 23.7**	**3.44**	**0.08**
PP × MLS	**1, 19.7**	**3.45**	**0.08**	1, 29.2	1.13	0.30	1, 28.9	0.56	0.46	1, 30.0	0.29	0.60	1, 27.8	0.07	0.79	1, 25.1	0.31	0.58
Second litter size		–			–		**1, 30.0**	**0.25**	**0.62**		–		**1, 29.0**	**1.42**	**0.24**	**1, 26.4**	**1.91**	**0.18**
Own litter size	**1, 28.1**	**0.04**	**0.84**		–			–		**1, 29.8**	**4.65**	**0.04**		–		**1, 22.4**	**3.52**	**0.07**
Log (body mass)		–		**1, 27.5**	**4.43**	**0.05**		–		**1, 30.9**	**14.3**	**<0.01**		–			–	
PP × OLS	**1, 17.6**	**7.30**	**0.02**		–			–			–			–			–	
MLS × SLS		–			–		**1, 26.0**	**3.97**	**0.06**		–		**1, 24.1**	**4.02**	**0.06**	**1, 21.7**	**3.80**	**0.06**

All items in bold are included in the final model and values reflect the output of the final model. Items not in bold were not included in the final model.

Urinary levels of 8-hydroxy-2-deoxy guanosine also did not vary with postpartum pregnancy or with manipulated litter size, and did not vary between the two urine collection points at the peak of the first and second lactations (PP: *F*_1, 30.6_ = 0.01, *P* = 0.94; MLS: *F*_1, 30.6_ < 0.01, *P* > 0.99; PP × MLS: *F*_1, 30.6_ = 0.04, *P* = 0.85; Time: *F*_1, 32.8_ = 1.19, *P* = 0.28).

### Reproductive outcomes

**Figure 3:**
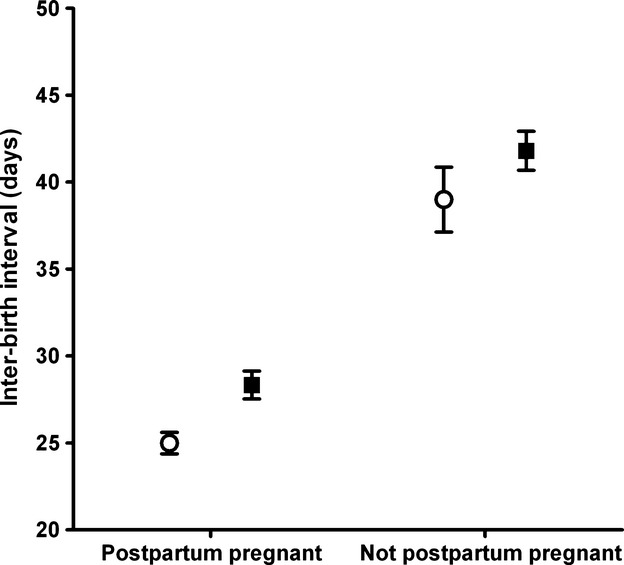
The interval between the first and second birth is longer for dams that were allocated litters of eight pups (closed squares) than dams that were allocated two pups (open circles) in the first lactation.

Interestingly, while we detected no consistent increases in oxidative stress with our gradient of reproductive effort, females exhibited changes in their reproductive scheduling that may have allowed the costs of reproduction to be offset. As an inevitable result of our manipulation, postpartum pregnant females displayed shorter intervals between the first and second parturition than females in the sequential breeding group (*F*_1, 14.7_ = 152, *P* < 0.01). However, females that were allocated a large litter also displayed longer inter-birth intervals than females that were allocated a small litter (*F*_1, 21.4_ = 8.24, *P* = 0.01, see Fig. 3; PP × MLS: *F*_1, 31.0_ = 0.01, *P* = 0.94). This effect was consistent among both postpartum pregnant and sequentially breeding dams and suggests that one of the costs of caring for large litters is a slower birth rate. Inter-birth interval was also negatively correlated with second litter size (*F*_1, 30.9_ = 5.45, *P* = 0.03, *R*^2^ (postpartum breeding) = 0.06, *R*^2^ (sequential breeding) = 0.13).

**Figure 4:**
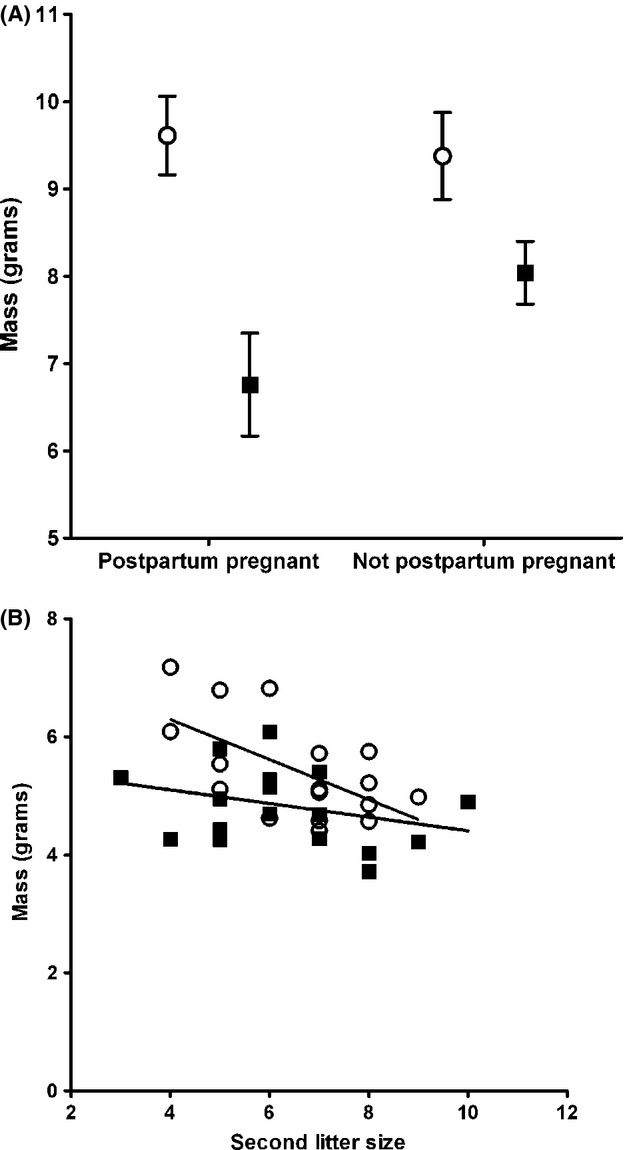
(A) The top panel depicts mean pup mass at the end of the first lactation. Pups from small litters (open circles) are heavier than those from large litters (closed squares), and pups from large litters and postpartum pregnant dams fare worst. (B) The second panel depicts mean pup mass at the peak of the second lactation. Pups from post-partum pregnancies (open circles) are heavier on average than pups from sequential litters (closed squares).

Neither of our manipulations produced observable differences in the number of pups born in the second litter, unlike previous studies conducted using laboratory strains of mice (Norris and Adams 1981; Johnson et al. 2001). However, they did cause differences in offspring performance over the course of the two lactations. In both litters, mean pup weight was driven by the postpartum breeding treatment and litter size. At 28 days old, pups from the first (manipulated) litter were lighter if they came from large litters, and the mass difference between pups from small and large litters was exaggerated if the dam was also postpartum pregnant, with pups from large litters and postpartum pregnant dams faring worst (PP: *F*_1, 25.5_ = 2.75, *P* = 0.11; MLS: *F*_1, 26.8_ = 23.4, *P* < 0.01; PP × MLS: *F*_1, 31.7_ = 5.00, *P* = 0.03, Fig. 4A). Pups from the second (unmanipulated) litter were also lighter if they came from large litters, but in contrast to the first litter, pups born from postpartum pregnancies were heavier on average (PP: *F*_1, 25.9_ = 6.64, *P* = 0.02; MLS: *F*_1, 24.1_ = 0.22, *P* = 0.64; PP × MLS: *F*_1, 28.2_ = 0.89, *P* = 0.35; SLS: *F*_1, 25.9_ = 14.1, *P* < 0.01; PP × SLS: *F*_1, 24.7_ = 3.65, *P* = 0.07, Fig. 4B).

## Discussion

Our manipulations of reproductive effort appeared to be effective in building a gradient of increasing energetic demands. Postpartum pregnant dams and those lactating for large litters ate more food, and females lactating for large litters exhibited longer inter-birth intervals than those lactating for small litters. The delay between births suggests a palpable life-history cost of lactating for experimentally enlarged litters. The increase in food consumption also hints at the costliness of lactation and postpartum pregnancy. While our upper-manipulation (large litters with concurrent pregnancy) is by no means unusual for a mouse, it is probably at the upper threshold of the reproductive costs that they would face naturally (Berry 1981) and the most demanding manipulation used in this context to date. And yet we found little support for our prediction that oxidative damage would be elevated with postpartum pregnancy or experimental enlargement of litters. Our results, when considered in relation to previous studies, further illustrate the absence of a simple relationship between oxidative stress and reproduction.

Our findings are consistent with the recent findings of Garratt et al. (2013) who also reported no increase in oxidative damage among females lactating for large litters. Instead, they documented an increase in the activity of the antioxidant enzyme superoxide dismutase in the livers of females lactating for large litters, which suggests that females made to increase reproductive investment also invested more in at least one aspect of antioxidant defence. In an earlier study, Garratt et al. (2011) also documented elevated concentrations of the antioxidant glutathione among reproducing female mice. The extent to which up-regulation of antioxidant defences occurs as a routine physiological change during reproduction is still unclear and warrants further investigation.

Despite failing to observe differences in oxidative damage between the groups, we did see evidence of indirect costs of reproduction, as defined by Hamel et al. (2010). The first of these costs was the longer delay between births among females that were allocated large litters in the first lactation. In this study, most postpartum pregnant animals delayed implantation, leading to inter-birth intervals well beyond the 19–21 days that characterise normal gestation (Berry 1970). For postpartum pregnant females, the longer inter-birth interval observed among dams that were lactating for large litters was the result of these individuals delaying the implantation of blastocysts in the second litter, and thus extending the period of gestation. We can draw this conclusion with confidence because the window in which a postpartum conception can occur is typically less than a day. Although positive correlation between suckling litter size and the duration of postpartum pregnancy has been observed previously (Mantalenakis and Ketchel 1966; Norris and Adams 1981; Johnson et al. 2001), this study is the first to our knowledge to apply consistent experimental manipulations to demonstrate this relationship. We have therefore shown that pregnant-lactating females will delay implantation for a longer period in direct response to a larger suckling litter.

Interestingly, dams that were not postpartum pregnant and were allocated a large litter also displayed longer inter-litter intervals than dams with small litters. It is impossible for us to know whether this delay was also due to delayed implantation, because we are not able to calculate the date of conception for these animals. It is perhaps more likely that this delay reflects differences in how soon dams with small and large litters wean their young and return to oestrus (and therefore variation in the date of conception), rather than variation in the period of gestation.

The second cost of reproduction was evident in the weight of pups from the manipulated litter, a potentially important component of offspring performance. Females lactating for small litters produced larger pups at 28 days old than females lactating for large litters. This is a well-established phenomenon (for example König et al. 1988), but was made more interesting by its interaction with the postpartum pregnancy treatment. The cost of lactating for large litters was compounded by postpartum pregnancy, insofar as pups from large litters whose mother was concurrently pregnant during lactation were smaller than pups whose mother was not pregnant. It seems that this added burden of postpartum pregnancy was not felt among postpartum pregnant dams lactating for small litters, whose pups were of a comparable weight to those of non-pregnant dams. König and Markl (1987) similarly reported that pups of postpartum pregnant dams were lighter at weaning than those of non-pregnant dams, although they did not investigate how this effect interacted with litter size. They suggest that females undergoing costly reproductive events will wean their young at the earliest possible state of physiological independence, even though a small body size at weaning can have serious fitness consequences, particularly for male offspring (Krackow 1993). In species like mice, where females have the capacity to produce many offspring during multiple reproductive events throughout their life, females may compromise their investment in particular offspring in order to protect their soma and improve their prospects for future reproductive bouts.

The costs of postpartum pregnancy evident throughout the first lactation were turned on their head during the second lactation, where we observed pups from postpartum pregnancies to be heavier at peak lactation than pups from sequential pregnancies. This effect has been reported in a previous study (Thompson and Anderson 1977), as has its reverse (Johnson et al. 2001). The vast array of inconsistent responses to postpartum pregnancies may reflect substantial differences between the strains of mice used in these studies. In this study we used mice that were first generation descendants from wild-caught mice. Thus the relationships seen in this population of mice are derived from selection on mice in the wild. However, the variable responses seen across types of mice highlight the complex nature of reproductive trade-offs and suggest that adaptive responses to costly reproductive events can be highly plastic and context-dependent.

Studies of the energetic demands of postpartum pregnancies have also produced inconsistent results, despite predictions that the period of overlap between the two litters should increase energetic expenditure and thus produce clear costs (Oswald and McClure 1990; Johnson et al. 2001). For example, Biggerstaff and Mann (1992) and Johnson et al. (2001) observed no increase in the food consumption of concurrently pregnant and lactating laboratory mice above levels observed in lactating females, while Krackow and Hoeck (1989) did find such an increase. Our observation that postpartum pregnant females ate more than non-pregnant lactating females may indicate that there are substantial differences between the energetic requirements of reproducing laboratory strains and wild mice, or that concurrently pregnant and lactating wild mice exhibit more potential for hypertrophy than do laboratory mice. Unfortunately, we only measured food consumption during a brief window and our measurements include the consumption of the males that were cohabiting with the lactating dams during this time, so our interpretation of differences in energy requirements between these groups is, of necessity, limited. Despite these limitations, the observed increase in food consumption is a tantalising hint that the energetic costs of postpartum pregnancy are substantial. This result calls for a more detailed investigation of the energetic costs of concurrent pregnancy and lactation in wild mice. This result also suggests that the costs of reproduction might be exposed more starkly under food limitation.

It is never possible to measure all possible components of parental investment, reproductive effort and metabolic response, especially without interfering with other traits. Nonetheless scope exists for studies that take other approaches to this question, including the measurement of milk yield and closer attention to food intake to provide different estimates of maternal allocation. Likewise, oxidative stress might manifest at times and in tissues other than those we studied. Perhaps terminating this experiment during peak reproductive investment would have provided a different result. But theories positing a role for oxidative stress in reproductive trade-offs do not predict that the stress will be so ephemeral as to disappear within days of reproduction.

Fletcher et al. (2012) found that wild lactating red squirrels (*Tamiasciurus hudsonicus*) displayed greater oxidative damage in serum than non-reproducing individuals, but in a group of animals that had food experimentally supplemented levels of oxidative damage were diminished. This reduction in serum oxidative damage was seen in all animals sampled in this food supplemented environment and suggests that access to abundant resources may help to reduce oxidative stress in wild animals. The authors also suggest that restricting an animal's access to food resources may help to reveal the effects of reproduction on oxidative stress (Fletcher et al. 2012). However, it is important to consider that animals in the wild often breed in situations where resources are abundant (Speakman and Krol 2010) and allocation to reproduction in mammals is known to be constrained, partly, by internal constraints that limit the amount of energy that can be metabolised (Speakman 2008), such as the ability to dissipate heat (Krol et al. 2007). When diets are restricted in some mammals, like mice, females will also simply reduce their litter size, usually by culling pups, or stop reproducing altogether, rather than go outside of their energy budget (Perrigo 1987; Speakman and Krol 2010). Thus, if oxidative stress is a very important physiological mediator of life history trade-offs, as hypothesised, it is perhaps surprising that we see no evidence of this when animals are reproducing at their upper-threshold, regardless of whether resources are limited or not.

In summary, we found little convincing evidence that increasing reproductive effort leads to greater oxidative damage. Instead, our study reveals physiological adaptations, such a delayed implantation, that allow females to offset concurrent periods of energetically costly reproduction, presumably to mitigate the increased costs to their soma and improve their future reproductive prospects. Although these life-history adjustments represent indirect reproductive costs, the physiological costs of reproduction that underlie the reproduction-longevity trade-off still remain elusive. Our study addresses one important recently highlighted limitation of previous studies testing for oxidative stress as a cost of reproduction – that those studied did not manipulate reproductive effort (Metcalfe and Monaghan 2013). We agree with additional suggestions that careful experimental studies are now required that limit food abundance whilst also manipulating reproductive investment (Selman et al. 2012; Metcalfe and Monaghan 2013).

## Data Accessibility

The authors intend to archive the data associated with this publication at Dryad (datadryad.org/). Data will be made publicly available and the location disclosed upon publication of this study.
